# Supplementary material for: Association between higher urinary normetanephrine and insulin resistance in a Japanese population
Source: PLoS One. 2020 Feb 13;15(2):e0228787. doi: 10.1371/journal.pone.0228787 (PMC7018048; doi:10.1371/journal.pone.0228787)
Supplement: Attachment — Submitted filename: PlosOne-NM-response to reviewers-2.docx [file pone.0228787.s001.docx]

Responses to Journal Requirements:

1. Please ensure that your manuscript meets PLOS ONE's style requirements, including those for file naming. The PLOS ONE style templates can be found at

<http://www.journals.plos.org/plosone/s/file?id=wjVg/PLOSOne_formatting_sample_main_body.pdf>and <http://www.journals.plos.org/plosone/s/file?id=ba62/PLOSOne_formatting_sample_title_authors_affiliations.pdf>

-----We ensure that our manuscript meets PLOS ONE's style requirements.

2. Please refer to any post-hoc corrections to correct for multiple comparisons during your statistical analyses. If these were not performed please justify the reasons.

Please refer to our statistical reporting guidelines for assistance (<https://journals.plos.org/plosone/s/submission-guidelines.#loc-statistical-reporting>).

----We wrote all statistical infromation in the text. In addition, we also added the statistical power caliculation in the text, upon the requirement from the reviewer 2 as described in “ responses to reviwer 2”

Responses to reviwers

Dear reviewer #1:

Thank you very much for having reviewed our manuscript entitled " Association between higher urinary normetanephrine and insulin resistance in a general population: PONE-D-19-29066". We have changed our manuscript to fulfill your criticisms as much as possible, and in most cases, I just changed as you requested. In any case, we beg your generosity to kindly feel satisfied with my responses. The details of the responses to each criticism were written below.

 This article is very interesting because authors investigated association between urinary metanephrine/normetanephrine and insulin resistance/secretion in a large cohort. I think this study has enough novelty and priority. Almost all of investigations and analyses were appropriate and performed adequately, but I would like to suggest authors to confirm some points.

-----I appreciate your evaluation.

1. Authors quoted various accurate references, particularly about the relationship between catecholamines and glycemic characteristics. However, I would like you to check one sentence, which was very important. Authors of ref. 17 revealed differences in the actions of adrenaline and noradrenaline with regard to glucose intolerance in patients with pheochromocytoma. In the article, authors of ref. 17 described “Regression analysis revealed that the improvement in HOMA-B from before to after surgery had a significant positive association with the improvement in urinary levels of metanephrine after surgery (P = 0.0286), and a significant negative association with the improvement in urinary levels of normetanephrine after surgery (P = 0.0248). The improvement in HOMA-IR did not show a positive association with the improvement in urinary levels of metanephrine but showed a significant positive association with the improvement in urinary levels of normetanephrine (P = 0.0001)” in Results of ref. 17. However, authors wrote “the improvement in U-M concentrations was "negatively" associated with the improvement in HOMA-B” (sentence 77-79). “the improvement in urinary metanephrine was “positively” associated with the improvement in HOMA-B” must be proper. In discussion, authors’ description was proper (sentence 229-232), so I think authors miswrote. Authors should check ref. 17 again, and then modify this description.

-----Thanks for your pointing. We correct the miswriting as you pointed.

2. As for description, authors must be more careful. Authors should modify below, and then check whole manuscript once more.
-----Thanks for your comments and I apologize such miswriting. We corrected as you suggested as follows.

Sentence 31 “normetanephrine (U-NM) and metanephrine (U-M) “should be modified to “urinary normetanephrine (U-NM) and metanephrine (U-M)”.

------Thanks. We corrected them as you mentioned.
Sentence 44 “HOMA-B” is incorrect, I think. Authors should modify this to “HOMA-R”.

------ Thanks. We corrected them as you mentioned.
Sentence 67 “throughα 1” should be modified to “through α1”.

------- Thanks. We corrected them as you mentioned.
Sentence 73 “normetanephrin” should be modified to “normetanephrine”.

------ Thanks. We corrected them as you mentioned.
Sentence 76 “changes in U-NM and U-MN concentrations” should be modified to “changes in U-M and U-NM concentrations”.

----- Thanks. We corrected them as you mentioned.
Sentence 212, 215, 219 Authors wrote “CA concentrations”, “CA concentration”, and “CAs concentrations” respectively. Authors should unify the writing.

------ Thanks. We unified the words as “CA concentrations”.

Dear reviewer #2:

Thank you very much for having reviewed our manuscript entitled " Association between higher urinary normetanephrine and insulin resistance in a general population: PONE-D-19-29066". We have changed our manuscript to fulfill your criticisms as much as possible, and in most cases, I just changed as you requested. In any case, we beg your generosity to kindly feel satisfied with my responses. The details of the responses to each criticism were written below.

I enjoyed reading this manuscript, in particular, the detailed analyses using appropriate statistical models, and the explanation of the results based on the analyses. However, I have some concerns.
-----Thanks for your evaluation.

(a) NO sample size/power statements are provided, and that needs to be justified wrt. the sample size of the analysis.

-----Thanks for your comment. We added such statement in “discussion” as follows: Contrary, adjustment for multiple factors can …….statistical power………..was 99.8% to detect an OR of 2.73 for insulin resistance at a significance level of 0.05, ………….

(b) Results on logistic regression should be reported in terms of effects sizes, p-values AND an indication of the uncertainty (typically, 95% confidence intervals, henceforth, CIs), given that p-values and CIs provide complementary information; see article <https://www.ncbi.nlm.nih.gov/pmc/articles/PMC2689604/>
Furthermore, results should be explained that way.

-----Thanks for your comments. We added p values for the analyses in “Results”.

(c) The title of the paper says "General population"; however, the target population is Japanese. This can be confusing to readers!, given that the authors are not consider a global population (spanning across all continents). The title should be revised.

-----Thanks for your comments. We corrected the title as follows:…in a Japanese population.
